# Multiconvolutional Transfer Learning for 3D Brain Tumor Magnetic Resonance Images

**DOI:** 10.1155/2022/8722476

**Published:** 2022-08-23

**Authors:** S. K. B. Sangeetha, V. Muthukumaran, K. Deeba, Hariharan Rajadurai, V. Maheshwari, Gemmachis Teshite Dalu

**Affiliations:** ^1^Department of Computer Science and Engineering, SRM Institute of Science and Technology, Chennai, India; ^2^Department of Mathematics, Faculty of Engineering and Technology, SRM Institute of Science and Technology, Kattankulathur 603203, Tamilnadu, India; ^3^School of Computer Science and Applications, REVA University, Bangalore 560064, India; ^4^School of Computing Science and Engineering, VIT Bhopal University, Bhopal-Indore Highway Kothrikalan, Sehore, MP, India; ^5^School of Information Technology and Engineering, Vellore Institute of Technology, Vellore, Tamil Nadu, India; ^6^Department of Software Engineering, College of Computing and Informatics, Haramaya University, POB 138, Dire Dawa, Ethiopia

## Abstract

The difficulty or cost of obtaining data or labels in applications like medical imaging has progressed less quickly. If deep learning techniques can be implemented reliably, automated workflows and more sophisticated analysis may be possible in previously unexplored areas of medical imaging. In addition, numerous characteristics of medical images, such as their high resolution, three-dimensional nature, and anatomical detail across multiple size scales, can increase the complexity of their analysis. This study employs multiconvolutional transfer learning (MCTL) for applying deep learning to small medical imaging datasets in an effort to address these issues. Multiconvolutional transfer learning is a model based on transfer learning that enables deep learning with small datasets. In order to learn new features on a smaller target dataset, an initial baseline is used in the transfer learning process. In this study, 3D MRI images of brain tumors are classified using a convolutional autoencoder method. In order to use unenhanced Magnetic Resonance Imaging (MRI) for clinical diagnosis, expensive and invasive contrast-enhancing procedures must be performed. MCTL has been shown to increase accuracy by 1.5%, indicating that small targets are more easily detected with MCTL. This research can be applied to a wide range of medical imaging and diagnostic procedures, including improving the accuracy of brain tumor severity diagnosis through the use of MRI.

## 1. Introduction

In the discipline of deep learning, mathematical optimization and pattern search are frequently employed to train computing models to learn from data and anticipate outcomes. Effective patterns for deep learning algorithms can be discovered and employed by using a set of labeled training data. Deep learning has attained cutting-edge performance for image recognition on difficult visual tasks [1]. In typical deep learning applications, extremely large datasets are utilized to provide the algorithm with a diverse set of examples, thereby enabling the algorithm to develop the ability to generalize to new, unexplored data. In general, the greater the amount of data available for a deep learning task, the greater the performance that can be achieved. However, in many fields, such as medical imaging, obtaining large, annotated datasets can be costly and difficult. In these types of applications, strategies that enable deep learning algorithms to use smaller datasets may enable previously unexplored important applications [2, 3].

Transfer learning refers to the reuse of weights from separate tasks as a baseline for the target application in order to speed up algorithm initialization and computation time. The use of transfer learning to alleviate some of the computational burdens of training can improve results and enable effective training of deep learning algorithms even with small datasets due to the high cost of data acquisition [4, 5]. In the field of brain tumors, where diagnosis based on traditional MRI can be difficult and requires the use of costly contrast injection procedures to better highlight the region of interest in the brain socket for the attending physician, this analysis can be of great benefit. We explore a method for transferring learned features from a much larger medical imaging database and applying them to a small dataset of unenhanced MR imaging of the brain to study this problem [6].

In addition, many types of medical imaging data can be extremely large and complex, with typical tissue slide images containing hundreds of millions of pixels with cellular resolution. Large structures, such as arteries, to small structures, such as nuclei, are depicted in these images [7]. When developing an algorithm to detect small abnormal tissue structures on images such as these, the number of negative or background regions will vastly outnumber the number of positive or diseased regions, and it can be challenging to receive extensive, detailed annotations that would require expensive physician effort [8]. Complex medical imaging datasets can be effectively analyzed with deep learning algorithms by utilizing a multi-resolution, hierarchical framework in which higher resolution regions of analysis are informed and determined by lower resolution structural region proposals, and this computation can be made more efficient through transfer learning from relevant domains.

The main contribution of the work is.

- To build a multiconvolutional transfer learning (MCTL) framework to examine and classify the dataset in a 3D imaging format, which, despite its additional complexity, can often contain important structural information with clinical relevance.
- Transfer learning initialization from a separate, larger, publicly accessible dataset will be compared to the random initialization.

Instead of relying on problem-specific heuristics or manually selected features, the proposed MCTL model and transfer learning implementation for training apply to other applications as well. It was discovered that the performance and robustness of models on this small dataset could be significantly enhanced by transferring knowledge.  Section 2 provides related work.  Section 3 presents a transfer learning method for better initialization, and a deep learning framework for assessing and identifying brain cancers in brain MRI.  Section 4 reviews the major contributions presented throughout the study, as well as potential avenues for future research in this field, while Section 5 provides a conclusion.

## 2. Background Study

Numerous real-world applications of computer vision, including text recognition, face recognition, mechanical inspection, robotics, and medical image analysis, have begun to make progress. Recent successful research in computer vision has relied heavily on deep learning to uncover complex hidden image representations. Small datasets, complicated images, and sparse labeling can all be challenges for deep learning in the field of medical imaging. In addition to providing background information on image analysis and deep learning methods, this section also discusses the specific medical imaging modalities—Magnetic Resonance (MR)—that are the subject of our current research as well as some of the deep learning innovations that have been made in these areas.

Multiple layers of simple patterns, referred to as neurons, are combined in CNNs to find complex patterns in images. The weights of filters that can characterize imaging data are learned and optimized rather than employing created features. Individual neurons in the visual field receive stimuli from their local regions and combine these signals to cover the full visual field, which was a major inspiration for the design of these devices [9]. As one of the deep learning's first and most successful uses, object classification in images is a key component of image recognition. ImageNet and other huge open-source datasets have aided in its quick advancement in the last several years. For image classification, AlexNet was one of the first popular deep learning frameworks [10]. It has 5 convolutional layers, followed by 3 fully connected layers, and at the time it was performed at the cutting edge on ImageNet [11, 12].

The term “object detection” refers to a technique for spotting specific types of semantic items in digital images. An object in a picture must not only be classified but it must also be located in the image. For this type of research, there are several significant open-source datasets available. One-stage frameworks and two-stage frameworks have been the two main pathways of deep learning research for object detection in recent years. It is possible to create regional ideas for possible object locations and then classify these regional proposals using two-stage frameworks. Utilizing the body's natural magnetic properties, MRI uses a strong magnetic field to produce an image of internal tissue [13, 14].

Hydrogen in the body has a relative “spin” vector that lines up in the direction of this magnetic field during imaging, which is abundant due to the high water content of the body. Pulsatile changes in the magnetic field vector cause atoms to align themselves with the new direction before returning to their resting state orientation [15]. The atoms emit a signal during this “relaxation” between pulses, which the MRI scanner's receiver coils can detect. The coils will track how long it takes for the protons to return to rest. Depending on the type of body tissue that is emitting the signal, this measurement can be affected by the concentrations of water and fat in the tissue. From any part of the body, the signal intensity can be monitored and used to create a sequence of cross-sectional images that show internal tissue in grayscale [16].

Imaging sources such as field intensity, radio frequency pulses, and receiver bandwidth can all contribute to MR images that are much noisier than other forms of imaging. The Rician distribution governs this noise because MR images are generated from the magnitude of complex number signal measurements [17]. Additionally, 3D image complexity is one of the most difficult aspects of analyzing MRIs in this work and previous studies. Additionally, the number of features that may be examined in a single image might grow exponentially, which can lead to inaccuracies in the registration of individual slices. Although MRI analysis has seen much advancement, the modality's strong sensitivity to detect sickness, such as malignant tumors, has led to many of these. In recent years, the emergence of multiple open-source MR imaging datasets has accelerated this advancement [18, 19].

Deep learning algorithm training can be time and resource-intensive. It can be difficult to prevent overfitting in many applications because the datasets are small compared to more standard deep learning applications. These problems can be overcome by training a model on a huge dataset from another application, then using the model as a feature extractor for learning in the target domain [20]. In medical imaging and other real-world applications, class imbalance in training datasets is a common problem. If this influence is not taken into account, a typical learner could end up favoring the majority or even completely overlooking the minority [21].

An inexpensive and minimally intrusive method to diagnose the severity of brain tumors using unenhanced MRI data can be applied to small datasets of unenhanced data, say researchers. There will be less work for doctors with this new technology, and patients will be spared another invasive contrast-enhanced imaging procedure, which is an added benefit [7, 22, 23].

Over the past few years, transfer learning has been thoroughly investigated, particularly in the area of computer vision, with several intriguing results. Transfer learning techniques have also been used in a number of researches to modify well-known networks to classify medical images. The VGG, AlexNet, or GoogleNet networks have been trained on the majority of cases. However, because the input size for these networks is fixed at 224*∗*224*∗*3, images must be reduced and their channels raised significantly to three before being supplied to the network. This method is ineffective and could damage the network's capacity for description. To this end, a better baseline method is necessary.

## 3. Multiconvolutional Transfer Learning (MCTL) Model

Medical imaging algorithms must have low error rates because of the seriousness of the consequences of a wrong categorization. It is common for early layers of deep neural networks to learn more universal features such as edges, which can be reused across different domains and enhance performance dramatically on very small datasets if they are removed from the dataset. If a method can improve diagnosis success rates for doctors who only use unenhanced brain tumor MRIs, the hospital's workflow can be improved while patients have a better imaging experience.

**Figure 1:**
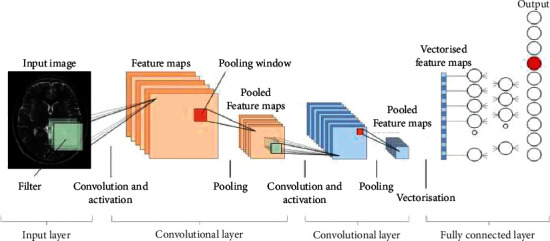
Multi-convolutional transfer learning (MCTL) architecture.

In order to detect brain cancer using MRI, this method contrasts models trained using weights taken from another medical imaging application with models trained with random initialization. On a short data set, the results are equivalent to those obtained by radiologists in practice. Finally, as seen in Figure 1, the method contrasts models trained using weights taken from another medical imaging application versus models trained with random initialization to detect brain cancer using MRI.

### 3.1. Preprocessing

Each MRI's 2D slices are used to create a 3D volume that can be used for training. In order for the relevant structures to be easily distinguished from the rest of the image, the image intensity window level has been set to cover 95% of the total voxel intensities in the image. Using the training dataset's mean brightness value, the algorithm can examine the relative contrast differences across photos, rather than the absolute brightness levels of each image. The range of the training dataset is used to normalize the brightness values. Images are classified as either healthy or unhealthy in this study using binary classification.

### 3.2. Data Augmentation

When possible, it is best to supplement a training dataset to improve the effectiveness of an algorithm in the form of adversarial networks. In order for this method to work effectively, a large amount of initial data is needed. In this study's perturbation approach of data augmentation, it was anticipated that each image would retain the same categorization regardless of how it was rotated and translated. In rotation, the position of an object in the frame is altered by randomly rotating a source image a certain number of degrees either clockwise or counterclockwise. Then, in order to adjust the image's new height and width, we get its new boundary dimensions from the sine and cosine of the rotation matrix. The matrix is then changed to take into account the height and width translation. then complete the affine transformation. It should be noted that the bounding box must be adjusted to take the result into account when object detection fails. An effective training set size of 18 can be achieved, providing the CNN with a large enough dataset from which to learn and classify the images. Each image is subjected to a total of 14 random operations, all of which involve the rotation and translation of individual pixels.

### 3.3. Multiconvolutional Layer

To assess these training data, a model of convolutional neural networks was created using a 3D picture as the input and a binary classification as the output. The model was constructed and trained using the Python Keras package. To find out how parameter choices and model designs affected performance, hyperparameter grid searches were done. The number of filters in each convolution layer could be changed from sixteen to sixty-four, and the number of convolutional layers could range from one to three (16, 32, or 64). The grid search is shown in Table 1. Extensive cross-validation was conducted for each combination of hyperparameters to determine how well the results were affected by changes in initial weights.

A flattened, fully linked layer, a binary classification output layer, and two 3D convolutional layers follow afterward. With a stride of 1, each convolution layer's kernels are 3 × 3 × 3 squares. The convolution weights are chosen according to a random distribution. In order to avoid vanishing gradient issues in deeper networks, the output is activated using a sigmoid function. Each intermediate layer employs ReLU activation functions. Using a binary cross entropy loss function, the model is optimized. We were able to choose a model by saving the network that had the highest accuracy in validation after each training stage. Nodes in the final layer of the model are used to calculate the expected probabilities of the two classifications. An image can be classified according to the output node that has the highest value when it is analyzed by a neural network. The disparity between the two output values increases as the model's confidence in its prediction increases. The main difficulty in using CNN models on limited datasets is overfitting.

### 3.4. Transfer Learning

**Figure 2:**
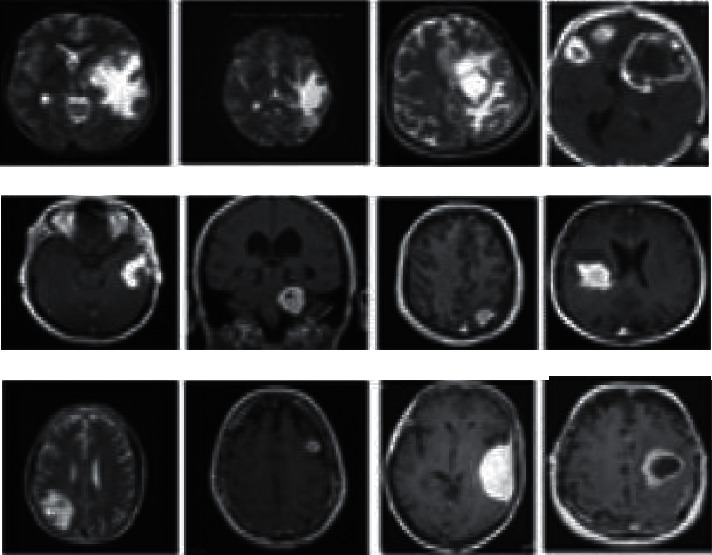
3D brain tumor MRI images of the REMBRANDT dataset.

An experiment that used convolutional features as an initialization for a brain tumor application was conducted after this. An excerpt from the REMBRANDT database was used to learn these features. The REMBRANDT dataset's 3D brain tumor MRI images are shown in Figure 2. The transfer learning process used a subset of the REMBRANDT database with 110020 images because the target dataset was T2-weighted. Large and complex MR images found in the REMBRANDT database necessitate problem-specific pre processes for obtaining local measurements.

**Figure 3:**
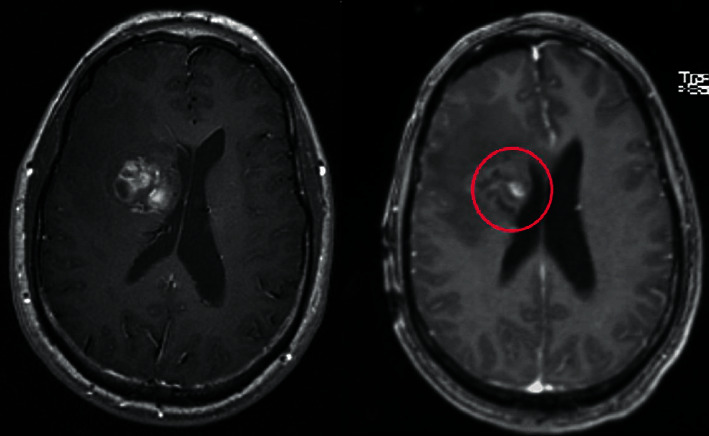
Autoencoder- output predictions.

Autoencoder-output predictions are shown in Figure 3. Each MRI yielded a dataset of 20000, 25 × 25 × 4 voxel patches, resulting in a total of 20000 voxels. The encoder portion's learned weights (“1,” “2,” and “3”) are used as initialization for the target classification model's convolutions during the transfer learning step, as shown in Table 2. Similar to models trained from random initialization, this one was also trained in the same way.

## 4. Results and Discussion

Convergence issues on a small data set can be addressed using transfer learning from a larger, 3D dataset. Algorithm predictions can also be sorted by model certainty in order to better match doctors with the cases that require their most attention. The model's output of classification certainty can be used to identify the majority of classification errors, which corresponded to the model's least certain predictions. Only cases with the most confident model output may be allowed to be classified, which may result in a large reduction in the workload of doctors and more time for them to concentrate on difficult cases.

When collecting unenhanced MRIs, the best model architecture created utilizing random initialization achieved an overall classification accuracy of 94 percent. It was expected that models with fewer trainable parameters would perform better in hyperparameter grid search because of the small size of the data set. When the number of layers and filters are equal, the models perform better with fewer nodes in a fully connected layer, a factor that significantly affects the number of trainable parameters. Convolutional filters have been added to each layer of transfer learning models to increase their accuracy and performance.

### 4.1. Dataset

Poonamallee private clinic provided a training dataset containing 50 unenhanced brains MRI studies for use in training. Data acquisition and analysis were conducted in accordance with all applicable guidelines and regulations. This information has been deemed exempt by the Government Medical Council Review Board. Prior to conducting this investigation, anonymized data were provided to the authors. Each MRI in this collection is from a patient who underwent an unexcited brain MRI and was between the ages of 18 and 45. The analysis employed a T2-weighted, fat-saturated picture from the coronal view of each MRI study since it had the highest sensitivity for the identification of brain tumors in any image.

### 4.2. Comparison Analysis

Brain MRI data were analyzed and a model was created and trained for identifying healthy and unhealthy samples. A leave-one-out cross validation (LOOCV) procedure was used to test this model. One LOOCV procedure can train 26 different models for a dataset of 45 patients. Each of these trained models had a 764-image training set, which included 32 synthetic augmentations for each sample. The model's overall performance can be better estimated by averaging the results of each LOOCV fold. A vast number of unique combinations of model parameter selections were subjected to a grid search in order to study the impact of hyperparameter selections and model designs on performance. For each set of hyperparameters, we performed a complete 15-time leave-one-out cross-validation to see how well the outcomes held up after the initialization weights were recalculated.

An average validation accuracy of 94 percent was achieved by the best-performing model, which had a specificity of 73 percent and a sensitivity of 85 percent. Predictions were ranked from least to most confident, and when the most confident predictions were included, the accuracy of the least confident predictions increased.  Table 3 depicts the average and comparison of various outcomes.

**Figure 4:**
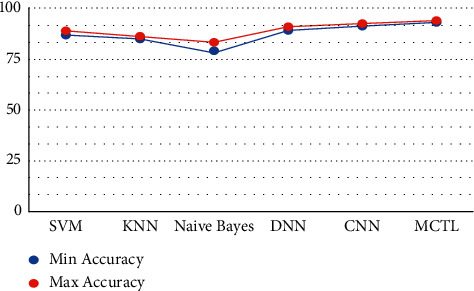
Accuracy.

**Figure 5:**
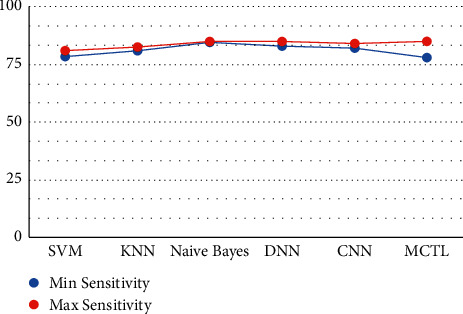
Sensitivity.

**Figure 6:**
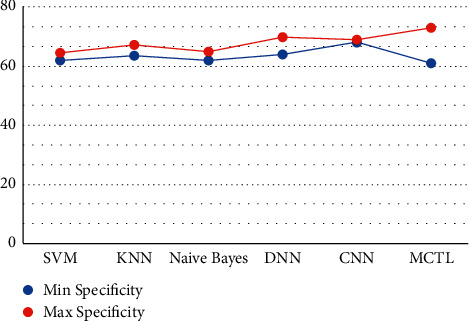
Specificity.

**Figure 7:**
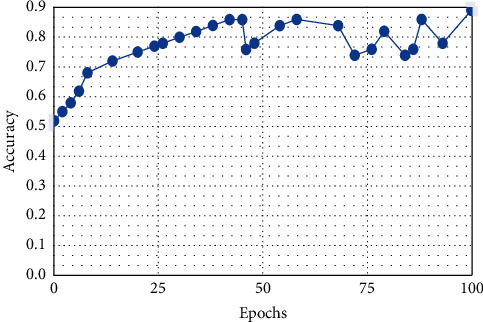
SVM.

**Figure 8:**
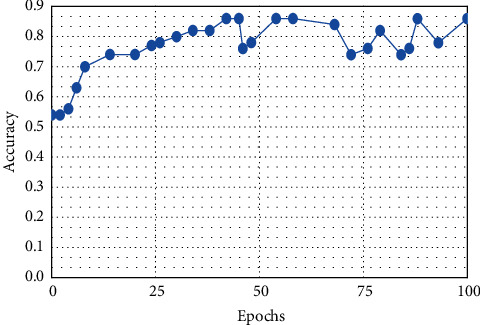
KNN.

**Figure 9:**
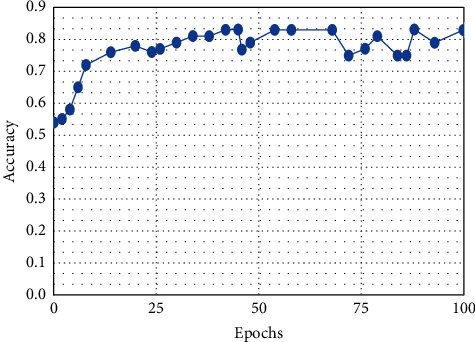
Naive bayes.

**Figure 10:**
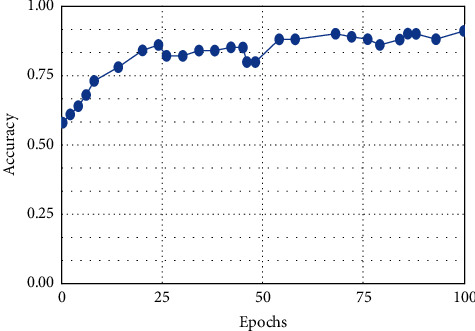
DNN.

**Figure 11:**
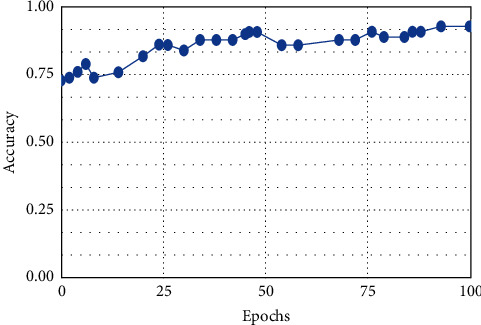
CNN.

**Figure 12:**
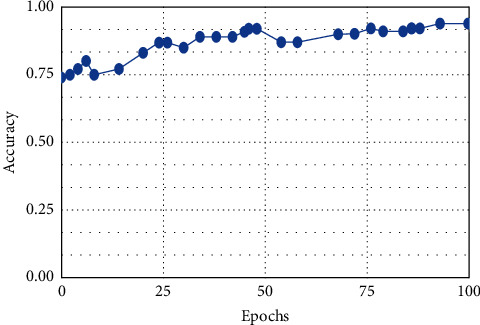
MCTL.

This result demonstrates that the proposed framework is attaining improved accuracy, sensitivity, and specificity with the same images, even though more data is required to validate this finding as shown in Figures 4–6 respectively. Performance in terms of brain tumor image classification was compared using accuracy. An epoch comparison of the existing inbuilt models [29] and the proposed model from Figures 7–11 show that using MCTL can improve the accuracy by 1.5%, thereby indicating that MCTL facilitates the detection of small targets shown in Figure 12.

## 5. Conclusion

This study examines deep learning for medical image categorization and applies it to the unenhanced MRI diagnosis of a brain tumor. A convolutional autoencoder is used to investigate a method for extracting relevant features from different 3D medical imaging datasets, and the results show that it performs better than random initialization. On a small dataset, achieving model convergence and accuracy provides hope that these methods can be used to analyze complex medical images. Although 3D imaging data is more difficult to analyze and classify than 2D data, it can contain important structural information that often has clinical significance. The multiconvolutional transfer learning (MCTL) framework based on convolutional neural networks was built to analyze and classify this data. Using MCTL has been shown to improve accuracy by 1.5%, indicating that small targets can be detected more easily with MCTL. Eventually, these methods will be applied to clinically relevant datasets as well. As well as improving the accuracy of brain tumor severity diagnosis through the use of MRI, this research can be applied to a wide range of other medical imaging and diagnostic procedures.

## Figures and Tables

**Table 1:** Proposed hyperparameter combination.

Convolutional layers	Filters	Nodes in fully connected layer

1	16	16, 32, 64
2	32	16, 32,64
3	64	16, 32, 64

**Table 2:** Proposed convolution layers performance.

No of convolution layers	Filters	Accuracy (%)	Sensitivity (%)	Specificity (%)
1	16	83–90	85–91	60–72
2	32	84–92	80–87	62–68
3	64	86–94	78–85	61–73

**Table 3:** Performance metrics - comparison analysis.

Reference	Method	Accuracy	Sensitivity	Specificity
[24]	Support vector machine (SVM)	87–89%	78.5–81%	62–64.5%
[25]	K nearest neighbor (KNN)	85–86.2%	81.1–82.6%	63.6–67.2%
[26]	Naive bayes	78.2–83.4%	84.8–85%	62–65%
[27]	Deep neural network (DNN)	89.3–91%	83–85$	64–69.8%
[28]	Convolutional neural network (CNN)	91.3–92.7%	82.2–84%	68–69%
	Proposed MCTL	93.2–94%	78–85%	61–73%

## Data Availability

The data used to support the findings of this study are included within the article.
